# Right Atrial Mass Discovered During Routine Follow-Up After Cardiac Procedures: Diagnostic Challenges and Management

**DOI:** 10.7759/cureus.64876

**Published:** 2024-07-18

**Authors:** Toshiaki Toyota, Tsuyoshi Suga, Taiji Okada, Atsushi Kobori, Yutaka Furukawa

**Affiliations:** 1 Department of Cardiovascular Medicine, Kobe City Medical Center General Hospital, Kobe, JPN; 2 Department of Diagnostic Radiology, Kobe City Medical Center General Hospital, Kobe, JPN

**Keywords:** antithrombotic therapy, thrombus, atrial septal defect closure, catheter ablation, atrial fibrillation, multimodal imaging

## Abstract

Cardiac masses pose significant diagnostic and therapeutic challenges in clinical practice. A 73-year-old male with a history of atrial fibrillation and percutaneous atrial septal defect (ASD) closure presented with an asymptomatic right atrial mass detected during routine transthoracic echocardiography follow-up. The mass measured 17 mm, with highly echoic peripheral areas and a heterogenous, low-echoic interior. The patient was asymptomatic and had no fever, embolic, or neurological symptoms. Multimodal imaging, including contrast-enhanced computed tomography, magnetic resonance imaging, and transesophageal echocardiography, revealed a mobile nodular mass in the right atrium (RA); however, the results of each modality were not consistently suggestive of a specific disease. The presumptive diagnosis of thrombus was made based on the change and variability of echocardiographic findings over time and the response to antithrombotic medications. Anticoagulant therapy with edoxaban led to the complete resolution of the mass, confirming the diagnosis of a thrombus. This case highlights the importance of multimodal imaging and temporal changes in findings in the diagnosis and management of RA masses and underscores the need for careful thrombotic risk assessment in patients with a history of atrial fibrillation, ASD, and cardiac procedures.

## Introduction

Cardiac masses pose significant diagnostic and therapeutic challenges in clinical practice. Although there are few and no coherent reports of cardiac mass, some follow a life-threatening course or serious complications [1]. Differential diagnoses include thrombi, tumors, and vegetation, and an accurate diagnosis is crucial for appropriate management and patient outcomes. This case report highlights the importance of multimodal imaging and the complexities involved in diagnosing right atrial masses, especially in patients with a history of cardiac procedures and atrial fibrillation.

## Case presentation

A 73-year-old male presented to a cardiology outpatient clinic for periodic examination. His medical history included hypertension, atrial fibrillation, atrial septal defect (ASD), and gastritis. He had undergone catheter ablation (pulmonary vein isolation and cavo-tricuspid isthmus (CTI) line) for atrial fibrillation three years prior and percutaneous ASD closure two years prior (Figulla Flex II, Occlutech International AB, Helsingborg, Sweden). Following these procedures, the patient underwent periodic transthoracic echocardiography (TTE). We performed his last examination three months prior, and no remarkable findings were observed after catheter ablation and ASD closure.

**Figure 1:**
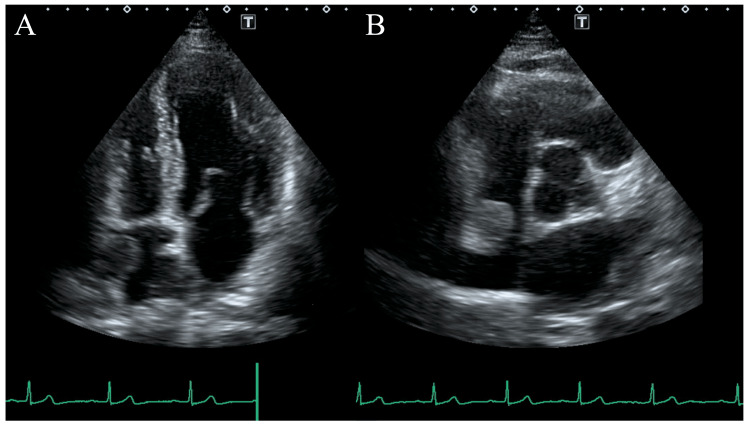
Transthoracic echocardiography. A) Four-chamber view. A round, rough surface mass was present in the right atrium; the area surrounding the mass was highly echoic, while the internal area was slightly low-echoic. B) Short axis view, aortic valve level.

TTE revealed a previously unobserved nodular mass in the right atrium (RA) (Figure 1). The mass measured 17×14 mm, with highly echoic peripheral areas and a heterogenous, low-echoic interior. Thus, a detailed examination of the patient was performed additionally. At presentation, the patient was asymptomatic without febrile symptoms or weight loss. Physical examination showed a blood pressure of 152/87 mmHg, heart rate of 64 beats per minute, body temperature of 36.4°C, respiratory rate of 14 breaths per minute, and oxygen saturation of 98% on room air. The neurological examination was unremarkable for the neurological diseases. Medication included amlodipine, azilsartan, and lansoprazole. Blood tests showed a C-reactive protein of 0.12 mg/dL, within normal limits. Chest radiography demonstrated a cardiothoracic ratio of 52% and no other particular findings. Electrocardiography revealed sinus rhythm.

**Figure 2:**
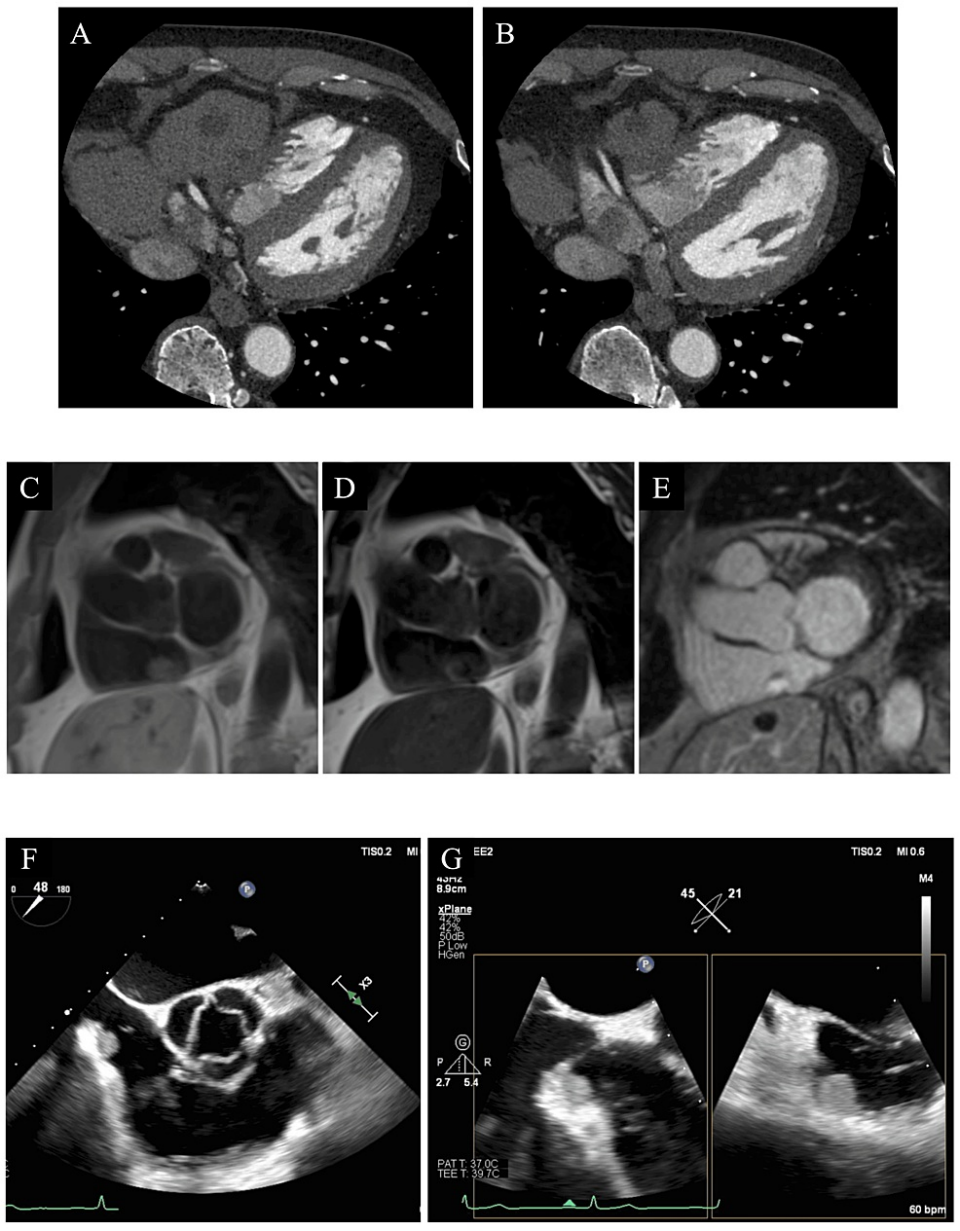
Multimodality assessment of the right atrial mass. A) CT, IVC level; B) CT, low right atrial level. Low CT value mass extending from the posterior wall of the right atrium; C) MRI, T1-weighted dark-blood; D) MRI, T2-weighted dark-blood; E) MRI, delayed gadolinium enhancement; F) transesophageal echocardiography, short axis view, aortic level; G) trans-esophageal echocardiography, x-plain view. CT, computed tomography; IVC, inferior vena cava; MRI, magnetic resonance image.

Contrast-enhanced computed tomography (CECT) revealed a 17-mm mass in the RA, hepatic cysts, and small cystic lesions in the pancreatic body and tail, with no other neoplastic lesions or enlarged lymph nodes, were observed. Magnetic resonance imaging (MRI) showed a mobile nodular lesion bordering the posterior wall of the right atrium with high intensity on T1-weighted images and low intensity on T2-weighted images, with gadolinium contrast enhancement in the surrounding area. Although there was a delay due to the coronavirus 2019 pandemic, transesophageal echocardiography (TEE) was performed two months after RA mass detection. TEE confirmed a high and modest echoic smooth mobile mass relative to previous TTE findings. The mass originated from a pouch-like lesion at the base of the eustachian ridge on the cavo-tricuspid isthmus line (Figure 2).

**Figure 3:**
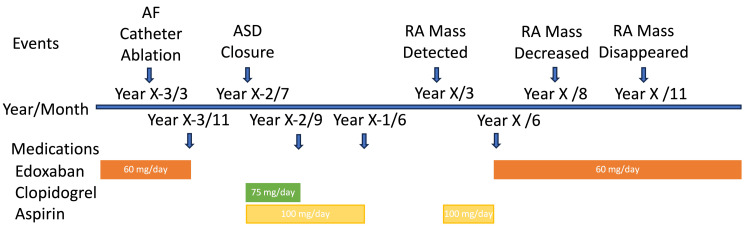
Time course of RA mass detection, cardiac procedures, and medications. AF, atrial fibrillation; ASD, atrial septal defect; RA, right atrium. Year X was defined as the year in which RA mass was detected.

Differential diagnoses included thrombus, fibroma, metastatic tumor, and sarcoma. His CHA2DS2-VASc score was 2, and his HAS-BLED score was 2 [2,3]. Direct oral anticoagulant therapy for atrial fibrillation and antiplatelet therapy following ASD closure had been discontinued 28 months and nine months prior to the RA mass detection, respectively. The D-dimer was not measured at the visit of RA mass detection; however, it was not elevated before and after the RA mass detection. In this case, we performed no antibody tests related to autoimmune diseases like antiphospholipid antibody syndrome. However, there was no history of embolism or coagulation abnormalities prior to this RA mass detection, and the physical examination findings were also negative for autoimmune diseases or coagulopathies. His family physician restarted aspirin treatment (100 mg/day) after the RA mass detection (Figure 3). After multimodality assessment and considering the temporal changes in echo findings of TTE and TEE, a presumptive diagnosis of thrombus was made. The patient discontinued aspirin and was prescribed oral edoxaban (60 mg/day). Follow-up CECT after two months from the initiation of direct oral anticoagulant therapy showed RA mass regression with no evidence of pulmonary embolism or systemic embolism. After five months of oral anticoagulant therapy, the mass was completely undetectable on both TTE and CECT (Figure 3). CECT confirmed the absence of tumors or embolisms. 

## Discussion

The main findings or learning points from this case are as follows: 1) The combination of TTE, CECT, MRI, and TEE is crucial for characterizing the RA mass and guiding its management. Each modality provides unique information that contributes to the diagnostic and treatment process; 2) The RA thrombus is rare but needs careful attention, especially in patients with atrial fibrillation and ASD, after catheter procedures.

Although we could not obtain tissue samples, the resolution of the RA mass after anticoagulant therapy confirmed the diagnosis of a thrombus, demonstrating the value of anticoagulation as both a diagnostic and therapeutic tool. At the time of RA mass detection, the MRI findings of high T1WI and low T2WI intensity with gadolinium enhancement and its rapid progression suggested a myxoma, fibroma, or sarcoma. Delayed enhancement is considered an atypical finding for thrombi, further complicating diagnosis. However, coincidentally delayed TEE findings and changes from the initial TTE increased the likelihood of a thrombus. MRI findings suggested the possibility of a benign tumor like myxoma or fibroma, but its rapid shrinkage without pulmonary embolism made this unlikely. Infective endocarditis was ruled out by the absence of fever or inflammatory reaction. In the present case, the D-dimer was negative before and after RA mass detection. Although the MRI results were not diagnostic in this case, MRI is generally useful for assessing the nature of the mass. We did not perform positron emission tomography-CT in this case; however, it helps diagnose the presence of malignancy and inflammatory disease. Still, in this case, we performed a systemic search with blood tests and contrast-enhanced CT and a diagnostic treatment with anticoagulant therapy, and the presence of malignancy or inflammatory disease was ruled out. Non-vitamin K oral anticoagulant was used instead of warfarin as the anticoagulant of choice because no findings in this patient suggested the presence of decreased renal function, post-mechanical valve replacement, or coagulation abnormalities such as antiphospholipid antibody syndrome. Multimodality findings with TTE, CECT, and TEE enabled monitoring of the response to therapy and confirming the diagnosis.

Reports on RA thrombus are limited. Preferential sites of intracardiac thrombi are often located within the left atrium, especially in patients with atrial fibrillation. In patients with impaired left ventricular function, thrombi are often found in the areas of reduced wall motion within the left ventricle. A case report of RA thrombus in a patient with recurrent classical Hodgkin lymphoma highlighted the importance of prophylactic anticoagulant therapy for an RA mass, which can lead to life-threatening complications [1]. A case report of a CTI thrombus detected during the catheter ablation procedure stated that the possible mechanism of the thrombus could be tissue overheating with energy application and coagulum formation, resulting in thrombus formation [4]. In contrast to a previous report, our patient had no RA mass three years after catheter ablation. The RA mass had originated at the site of CTI ablation; however, the ablation procedures were completed smoothly without complications, and the ablated tissue would have already healed within several months. During the double or single antiplatelet therapy periods, TTE could not detect the RA mass. Pouch-like lesions at the base of the eustachian ridge on the CTI would have acted as a blood pool where the blood flow was stagnant. Additionally, impaired right heart function after the long-term burden of ASD might exacerbate the thrombotic status of the patient.

The mass appeared after discontinuation of prophylactic anticoagulation or antiplatelet therapy after catheter ablation and ASD closure, highlighting the importance of reassessing the thrombotic risk in patients with a history of atrial fibrillation and ASD. A low CHA2DS2-VASc score and the location of the mass away from the ASD closure device could have kept physicians away from the diagnosis of thrombosis. This case emphasizes the need to consider thrombus formation even in low-risk scenarios. A meta-analysis suggested that it may be safe to discontinue oral anticoagulant therapy after successful ablation, even in patients with elevated CHA2DS2-VASc scores [5]. Additionally, a large Japanese cohort study found that continuing oral anticoagulant therapy beyond six months was associated with lower thromboembolism risk in patients with a CHADS2 score ≥3 but higher bleeding risk in those with a CHADS2 score ≤2 [6]. Additionally, the incidence of thrombus formation on septal closure devices is low but exists [7]. Thrombi may also be coincident with the patients with ASD device, thus antiplatelet therapy for at least six months after ASD closure is recommended [8]. In the present patient, we found an RA thrombus at a distance from the septal closure device. Patients with atrial fibrillation or ASD may have impaired atrial function, and patients with impaired atrial function can be regarded as a high-risk thrombotic group. In patients with suspected reduced atrial function, speckle tracking echocardiography would have an incremental diagnostic value in detecting atrial cardiomyopathy [9]. Although assessing right heart function in daily practice is not popular, it may be effective in patients who discontinue antithrombotic therapy after catheter ablation or transcatheter ASD closure. Additionally, the beginning of the RA thrombus is unclear because the patient had no symptoms. Only the imaging modality could detect it; however, judging from the TTE one year before the RA mass detection, there was no mass in the RA. If we cease antithrombotic therapy in patients with thrombotic risk, we might have to check the imaging follow-up more shortly. Considering the patient's clinical course, the anticoagulation therapy will be continued for as long as possible.

This case highlights the diagnostic challenges posed by right atrial masses and the importance of a comprehensive multimodality imaging approach. This underscores the need for clinicians to maintain a high index of suspicion for thrombi even in patients with atypical presentations or seemingly low-risk profiles. Successful resolution of the mass with anticoagulation therapy emphasizes the value of this approach in both diagnosis and treatment. Regular follow-up imaging is essential to monitor treatment response and confirm the diagnosis in such cases.

## Conclusions

Right atrial masses require multimodal imaging for accurate diagnosis and treatment. This case emphasizes the importance of considering thrombi, even in atypical presentations, and demonstrates the diagnostic and therapeutic value of anticoagulation in managing suspected cardiac thrombi.
